# Cyclin-dependent kinase 1 depolymerizes nuclear lamin filaments by disrupting the head-to-tail interaction of the lamin central rod domain

**DOI:** 10.1016/j.jbc.2022.102256

**Published:** 2022-07-15

**Authors:** Soyeon Jeong, Jinsook Ahn, Inseong Jo, So-Mi Kang, Bum-Joon Park, Hyun-Soo Cho, Yong-Hak Kim, Nam-Chul Ha

**Affiliations:** 1 Department of Agricultural Biotechnology, Center for Food and Bioconvergence, and Research Institute for Agriculture and Life Sciences, CALS, Seoul National University, Seoul, Republic of Korea; 2 Department of Molecular Biology, College of Natural Science, Pusan National University, Busan, Republic of Korea; 3 Department of Systems Biology and Division of Life Sciences, Yonsei University, Seodaemun-gu, Seoul, Republic of Korea; 4 Department of Microbiology, Catholic University of Daegu School of Medicine, Daegu, Republic of Korea

**Keywords:** lamin, nuclear lamina, phosphorylation, nuclear envelope, CDK1, mitosis, CDK, cyclin-dependent kinase, CK1, casein kinase 1, GSK3β, glycogen synthase kinase 3β, GST, glutathione-*S*-transferase, IF, intermediate filament

## Abstract

Nuclear lamins maintain the nuclear envelope structure by forming long linear filaments *via* two alternating molecular arrangements of coiled-coil dimers, known as A11 and A22 binding modes. The A11 binding mode is characterized by the antiparallel interactions between coil 1b domains, whereas the A22 binding mode is facilitated by interactions between the coil 2 domains of lamin. The junction between A11- and A22-interacting dimers in the lamin tetramer produces another parallel head–tail interaction between coil 1a and the C-terminal region of coil 2, called the ACN interaction. During mitosis, phosphorylation in the lamin N-terminal head region by the cyclin-dependent kinase (CDK) complex triggers depolymerization of lamin filaments, but the associated mechanisms remain unknown at the molecular level. In this study, we revealed using the purified proteins that phosphorylation by the CDK1 complex promotes disassembly of lamin filaments by directly abolishing the ACN interaction between coil 1a and the C-terminal portion of coil 2. We further observed that this interaction was disrupted as a result of alteration of the ionic interactions between coil 1a and coil 2. Combined with molecular modeling, we propose a mechanism for CDK1-dependent disassembly of the lamin filaments. Our results will help to elucidate the cell cycle–dependent regulation of nuclear morphology at the molecular level.

Intermediate filaments (IFs) form a robust protein network in the cytoplasm of most cells to provide mechanical strength to the cells (1, 2). All IFs share a typical structural organization, although they are substantially diverse in size and amino acid sequence. IFs can be divided into cytosolic and nuclear families by the features of the primary structures. All IFs consist of the central ⍺-helical rod domains, N-terminal head, and C-terminal tail regions (3, 4, 5). The central ⍺-helical rod domains are responsible for forming the parallel coiled-coil dimer because of its characteristic periodicities in the sequence. The central ⍺-helical domains are divided into several subdomains: coil 1a, linker L1, coil 1b, linker L12, and coil 2. The periodicities in the coil 2 region are different in the regions: hendecad repeats (residues 241–277), heptad repeats (278–319), stutter (residues 320–330), and heptad repeats (residues 331–385) (Fig. 1*A*) (6, 7, 8).

**Figure 1:**
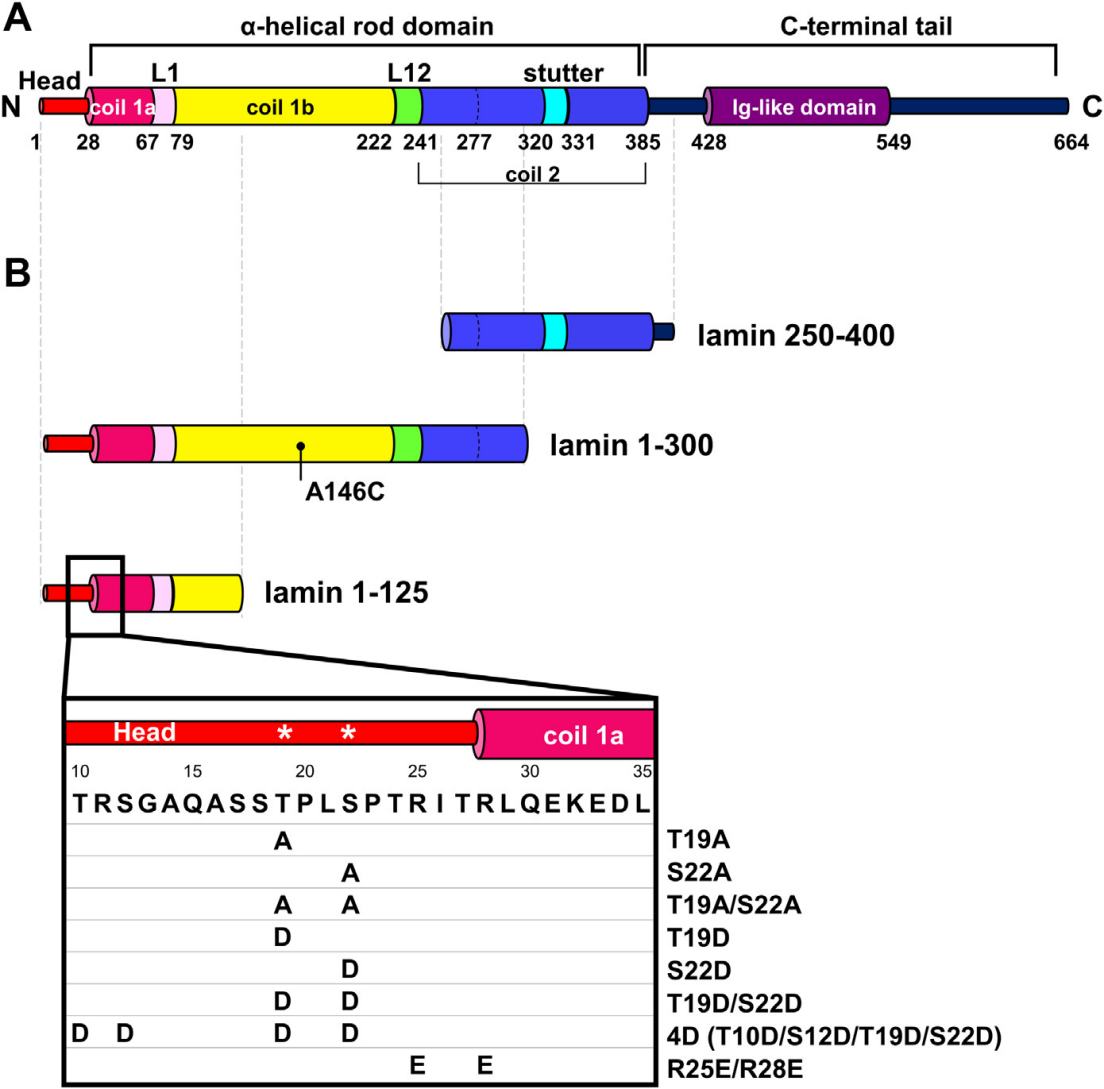
**The structural organization of lamin A/C and the lamin fragments used in this study.***A,* schematic diagram of the organization of lamin A/C, including the N-terminal head, the central ⍺-helical rod, and the C-terminal tail domains. The subdomains coil 1a, linker L1, coil 1b, linker L12, coil 2, stutter, and coil 2 are labeled in the ⍺-helical rod domain. The C-terminal tail contains the immunoglobulin (Ig)-like domain (*violet*). The unstructured regions are in the thinner cylinders. *B,* the lamin fragments used in this study: lamin 250 to 400, lamin 1 to 300, and lamin 1 to 125 fragments. The amino acid sequence of residues 10 to 35 is shown in the enlarged images in the *box*. The CDK1 phosphorylation sites are indicated by *asterisks*. The mutants used in this study are indicated in the corresponding positions. CDK1, cyclin-dependent kinase 1.

The nuclear IF lamins form a robust meshwork structure near the inner nuclear membrane inside the nucleus, primarily maintaining the nuclear envelope structure (9, 10, 11, 12). The nuclear IF lamin A/C is expressed in differentiated cells, unlike lamin B1 and B2, which are ubiquitously expressed in eukaryotic cells (13, 14). *In situ* cryo-EM images of the lamin A/C filaments have revealed that the thinner 3.5-nm-thick filament structure is distinct from the cytosolic IFs that exhibit the typical 10-nm-thick filament (11). Four types of dimer-to-dimer interaction modes (A11, A22, A12, and ACN) have been observed in IFs when forming the mature filament (6, 15, 16). The lamin filament model was proposed using three types of interaction modes (A11, A22, and ACN) based on the electron cryotomography and chemical cross-linking data (7, 17, 18, 19). They further proposed that four or more units of the 3.5-nm-thick filaments are laterally assembled into 10-nm-thick filaments of cytosolic IFs in the A12 interaction mode.

The structure of the long lamin A/C fragment (residues 1–300) has been shown to have an antiparallel arrangement of two parallel coiled-coil dimers at 3.2 Å resolution (7). This antiparallel arrangement of lamins was designated as the “A11” interaction, previously named because of the antiparallel interaction between the coil 1b regions from the adjacent coiled-coil dimers (1, 17). The A11 tetramers, formed by the A11 interaction, are further joined to elongate the linear filament by another interaction mode, A22, representing an antiparallel arrangement between the coil 2 regions (15, 20). The interaction mode, called ACN, represents a head-to-tail arrangement between coil 1a and the C-terminal region of coil 2. The sophisticated cross-linking mass analysis and modeling studies further suggested that the A22 and ACN interaction modes consequently indicate the same arrangement of the A11 tetramers. The A22 or ACN binding modes include not only the interaction between the coil 2 (referred to as A22 interaction) but also the coil 1a and the C-terminal region of coil 2 (referred to as ACN interaction) are formed (16, 17, 18).

The disappearance of the nuclear envelope is the hallmark of mitosis in eukaryotic cells (21, 22, 23). A critical feature of nuclear lamins is cell cycle–dependent polymerization and depolymerization, which determines the morphology of the nuclear envelope. To date, 92 phosphorylation sites that mainly concentrate in the head and tail domains of lamin A/C have been identified in lamin A/C throughout the cell cycle (9, 24). The activated cyclin-dependent kinase 1 (CDK1)–cyclin B complex at the onset of mitosis phosphorylates the lamin filaments, leading to the depolymerization of the filaments and a breakdown of the nuclear envelope (25, 26, 27). Phosphorylation at Thr19 and Ser22 in the lamin N-terminal region by the CDK1–cyclin B complex is known as the critical step for cell cycle–dependent disassembly of the lamin filamentous structure (26, 28). The mutation at Ser22 to Leu was associated with dilated cardiomyopathy (29). However, its phosphorylation-dependent lamin disassembly mechanism is poorly understood at the molecular level. In this study, we investigated the mechanism by which phosphorylation acts on the molecular interactions in the 3.5-nm-thick filament of lamin proteins.

## Results

### The phosphorylation of lamin by CDK1 inhibits the A22 interaction

To examine whether phosphorylation at the N-terminal fragments affects the A11-type interaction, we performed an *in vitro* binding assay using the lamin 1 to 300 fragment (residues 1–300) harboring the A146C mutation (Fig. 1*B*) (7). The lamin 1 to 300 fragment contains the head region (residues 1–27) and the central rod domain, which forms A11 tetramers without the A22 interaction. The disulfide bond between two coiled-coil dimers would be created within the A11 tetramer since Ala146 is in the center of the A11 tetramer, as confirmed in the crystal structure (7). Thus, we monitored the propensity of the A11 interactions as the amount of the disulfide forms by utilizing the A11-tetramer–dependent disulfide bond formation of the A146C mutant protein (7).

**Figure 2:**
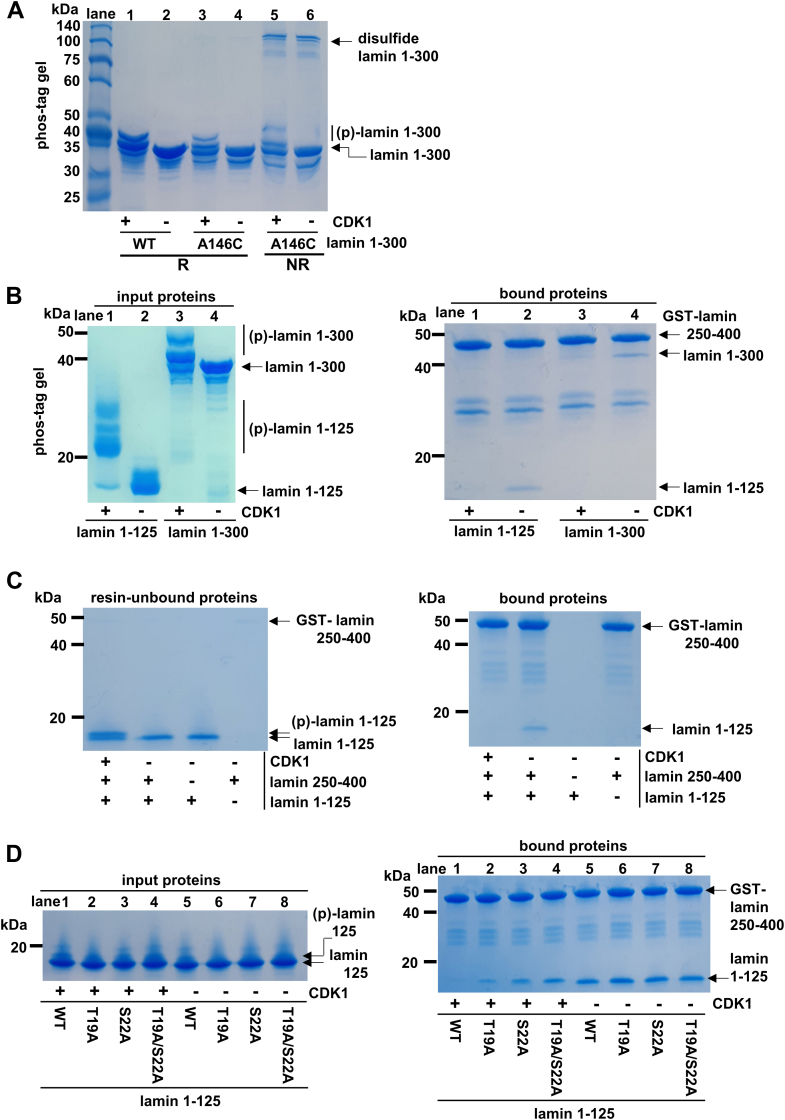
**The effects of CDK1–cyclin B1 activity on lamin proteins in the A11 and A22 interactions.***A,* monitoring the A11 interaction by the phosphorylation of the lamin 1 to 300 fragment by the CDK1 complex. The WT and A146C mutant lamin 1 to 300 fragments were phosphorylated by the CDK1 complex and then analyzed by a Phos-tag–containing SDS-polyacrylamide gel using reducing loading dye (R) or nonreducing loading dye (NR). The phosphorylated lamin 1 to 300 ((p)-lamin 1 to 300) fragment moved slower than the unphosphorylated lamin 1 to 300 in the Phos-tag–containing gel. Note that the disulfide-bonded lamin 1 to 300 fragment (disulfide lamin 1–300) formed regardless of the CDK1-mediated phosphorylation. *B,* phosphorylation of lamin N-terminal fragments (lamin 1–125; lamin 1–300) by the CDK1 complex and binding to the GST-fused lamin coil 250 to 400 (residues 250–400), which represent the ACN interaction. The input proteins for the binding assay are on the *left* Phos-tag–containing gel, and the proteins bound to the GSH-coupled resins are on the *right* SDS-polyacrylamide gel. The standard size markers are in the *left lane* in each gel. *C,* dissociation of the lamin complex by CDK–cyclin B activity. The resin of the lamin complex, consisting of lamin 1 to 125 and lamin 250 to 400 fragments, was incubated with the CDK1–cyclin B complex at room temperature. The resins were washed, and then the bound proteins were analyzed by SDS-PAGE. *D,* the amount of phosphorylation of WT or mutant lamin 1 to 125 is exhibited by SDS-PAGE (the *left panel*), and the amounts of the various mutant lamin 1 to 125 fragments bound to the GST-fused lamin 250 to 400 represent the ACN mode (the *right panel*). Before the binding assay, the lamin 1 to 125 fragment was phosphorylated by the CDK1 complex (lanes 1–4). The mutations are depicted at the *bottom* of Figure 1*B*. CDK1, cyclin-dependent kinase 1; GST, glutathione-*S*-transferase.

We phosphorylated the A146C mutant lamin 1 to 300 fragments with the CDK1–cyclin B complex. To distinguish the phosphorylated bands, we used a Phos-tag containing SDS-PAGE, in which the Phos-tag retarded the mobility of the phosphorylated proteins by the specific interaction with the phosphate group (Fig. 2*A*, (p)-lamin 1 to 300 in lanes 1 and 3) (30). These results confirm that the CDK1–cyclin B complex phosphorylates the lamin 1 to 300 fragments. We noted the amounts of the disulfide form of lamin 1 to 300 (A146C) under nonreducing conditions, which reflects the propensity of A11 tetramer formation. Similar amounts of disulfide forms were observed between the nonphosphorylated and phosphorylated lamin proteins. These results indicate that the A11 interaction is not significantly affected by the phosphorylation of the N-terminal head region by the CDK1–cyclin B complex (Fig. 2*A*, disulfide-bonded lamin 1–300 in lanes 5 and 6).

We next tested whether phosphorylation by the CDK1–cyclin B complex affects the A22- or ACN-type interaction. To monitor and distinguish the ACN mode and A22 interactions, we used two lamin N-terminal fragments and one lamin C-terminal fragment. For lamin N-terminal fragments, we used the lamin 1 to 125 (residues 1–125) and lamin 1 to 300 fragments. For the C-terminal lamin fragment, we used a lamin 250 to 400 fragment (residues 250–400), which spans the most part of the entire coil 2 (residues 242–385) and a C-terminal flanking region (residues 386–400). The lamin 250 to 400 fragment contains the structural elements for ACN mode, which is consistent with the after-stutter region composed of the heptad repeat periodicity (residues 331–385, Fig. 1*A*). Since the lamin 1 to 125 fragment does not contain the coil 2 region for the A22-type interaction, this fragment can detect only the ACN mode and not the A22 interaction. In contrast, the lamin 1 to 300 fragment could participate both in the A22 interaction and the ACN mode.

We first confirmed that both the lamin 1 to 125 and 1 to 300 fragments were phosphorylated well by the CDK1–cyclin B complex (the upshifted bands for the lamin 1–125 fragment in Fig. 2*B*, *left lanes* 1 and 3). In a glutathione-*S*-transferase (GST) pull-down assay to test the ACN and A22 interaction modes, we found that phosphorylated lamin N-terminal 1 to 125 and 1 to 300 fragments were weaker bindings to GST-fused lamin C-terminal 250 to 400 fragment than the unphosphorylated lamin N-terminal fragments. These observations showed that phosphorylation at the lamin N-terminal head region weakened the interaction with the lamin coil 2 region (Fig. 2*B*, *right lanes* 1 and 3). Since the phosphorylation of lamin influenced the interaction with the lamin 1 to 125 fragment that cannot participate in the A22 interaction, our findings further showed that phosphorylation is involved in the ACN interaction but not in the A22 interaction.

We next tested whether phosphorylation induces the dissociation of the complex in a reasonable time. We first prepared the lamin complex of the lamin 1 to 125 fragment and the lamin 250 to 400 fragment in GSH-coupled resin. Then, we incubated the CDK1–cyclin B complex with the lamin complex bound to resin for 2 h to allow the CDK1 enzyme to phosphorylate the lamin complex. The lamin complex was phosphorylated by the CDK1 enzyme as efficiently as the individual lamin fragment proteins. These results demonstrate that the CDK1 enzyme acts on the lamin complex to induce the dissociation of the lamin complex on a feasible time scale (Fig. 2*C*).

### Phosphorylation of Thr19 and Ser22 inhibits the ACN mode

The Thr19 and Ser22 residues in the lamin N-terminal head region are the target sites of the CDK1–cyclin B complex in the depolymerization of lamin filaments during mitosis (28, 31, 32). To determine which residues are more important in regulating the ACN mode dependent on CDK1 activity, we created single and double mutants of the lamin 1 to 125 fragment at Thr19 and Ser22: T19A, S22A, and T19A/S22A. The phosphorylation of the mutant lamin fragments by the CDK1 complex was assessed by mobility shifts on Phos-tag PAGE (Fig. S1). The double mutant T19A/S22A protein underwent a band shift, indicating the other CDK1 target sites in the lamin fragment containing residues 1 to 125.

The mutant lamin 1 to 125 fragments participated in the ACN mode as robustly as the WT fragment (Fig. 2*D*, the *right panel*, lanes 5–8), indicating that Thr19 and Ser22 are not involved in mediating the ACN interaction. However, when the lamin 1 to 125 fragments were phosphorylated by CDK1, the mutant lamin 1 to 125 fragments retained the ACN with the C-terminal fragment of lamin A/C coil 2, unlike the WT fragment (Fig. 2*D*, the *right panel*, lanes 1–4). When phosphorylated, the double mutant T19A/S22A protein showed the more evident effect than the single mutant proteins T19A and S22A. Thus, our findings suggest that phosphorylation at Thr19 and Ser22 in the lamin N-terminal region cumulatively contributes to the depolymerization of the lamin filaments. These results are consistent with the previous results for Thr19 and Ser22 in the cell cycle–dependent depolymerization of lamin filaments (28).

### The phosphorylation of lamin A/C does not affect the coiled-coil structure of coil 1a

Then, how does phosphorylation in the N-terminal head region abolish the ACN interaction? To answer this question, we first investigated the phosphorylation effects on the propensity for coiled-coil formation (Fig. S2). We noted the coil 1a region, which is adjacent to the phosphorylation sites because the coiled-coil tendency of the coil 1a region was reversely correlated to the ACN interactions in the L59R mutant (33, 34).

**Figure 3:**
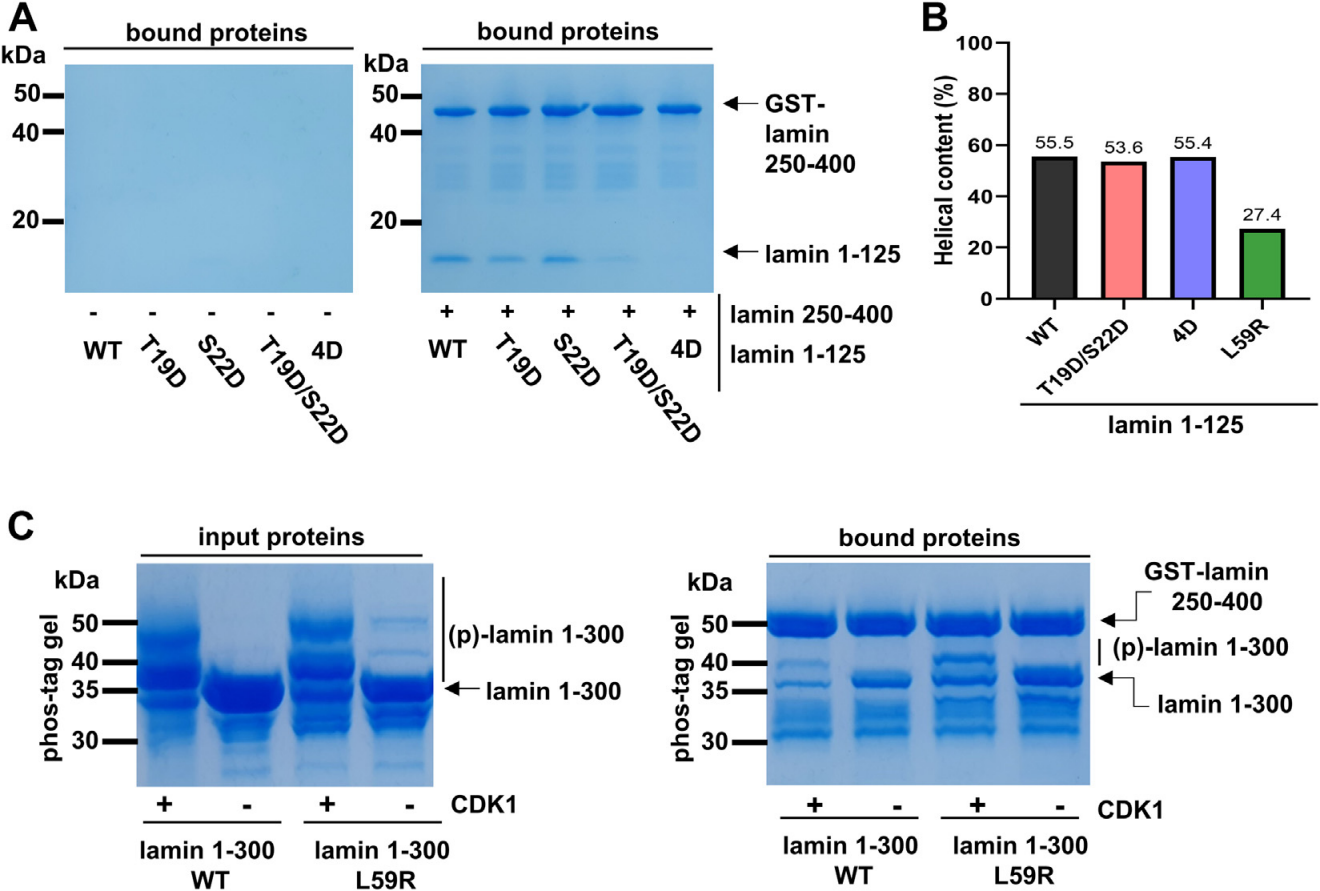
**Analysis of the coiled-coil propensity change of the coil 1a region because of CDK1 activity.***A,* GST pull-down assay between GST-fused lamin 250 to 400 and the mutant proteins of the lamin 1 to 125 fragment. The lamin 1 to 125 fragment mutations are labeled under the lane. “4D” stands for the quadruple mutation T10D/S12D/T19D/S22D. *B,* the helical contents from the CD spectra (Fig. S3) of the WT and mutant lamin 1 to 125 fragments. The portions of the secondary structure elements were analyzed by the CDNN software (43). *C,* GST pull-down assay between GST-fused lamin coil 250 to 400 and WT or L59R mutant lamin 1 to 300 fragments on a Phos-tag–containing SDS-polyacrylamide gel. The (un)phosphorylated WT and L59R lamin 1 to 300 fragments were prepared for the GST pull-down assay (input proteins). The phosphorylated lamin 1 to 300 ((p)-lamin 1 to 300) was upshifted on the gel (CDK1 + lanes). The (un)phosphorylated proteins were loaded on the GST-lamin coil 250 to 400–bound resin and then analyzed by the Phos-tag–containing SDS-polyacrylamide gel (bound proteins). CDK1, cyclin-dependent kinase 1; GST, glutathione-*S*-transferase.

We examined whether the phosphorylation of the lamin 1 to 125 fragments affected the coiled-coil propensity of the coil 1a region based on the CD results with the purified lamin fragments. This experiment used the phosphorylation-mimicking dual mutant T19D/S22D and quadruple mutant T10D/S12D/T19D/S22D (designated the 4D mutant in this study) instead of the phosphorylated lamin fragment. The dual and quadruple mutants were employed in this CD experiment because we could not obtain enough phosphorylated protein samples for the CD analysis. Phosphorylation at Thr10 and Ser12 was observed in the cellular lamin proteins, although the responsible kinases were unknown. The 4D mutation completely abolished the ACN between lamin 1 to 125 and lamin 250 to 400 fragments, whereas the ACN interaction was partially retained in the T19D/S22D mutant (Fig. 3*A*). Although the dual and quadruple mutations did not completely mimic phosphorylation by the CDK1 complex, our findings suggest that the charge introduction is essential in inhibiting the ACN mode without changing the coiled-coil propensity of the lamin 1 to 125 fragments (Figs. 3*B* and S3).

The genetic L59R mutation significantly weakened the helical propensity in lamin 1 to 125 fragment (33) (S3). The lamin aggregates were deposited near the perinuclear regions in the cells expressing the L59R mutant lamin A/C (7). Thus, we investigated the L59R mutation in terms of phosphorylation at the N-terminal head region of lamin. When we performed the ACN mode assay using the L59R-harboring lamin 1 to 300 fragments, the phosphorylation at the N-terminal head region of the lamin fragment did not abolish the ACN interaction, unlike the WT lamin fragment. These findings suggest that the genetic mutation L59R overwhelms the CDK1 function of the phosphorylation-dependent perturbation of the ACN interactions in the lamin filaments, which may explain the perinuclear deposition of the lamin filaments by the mutation (Fig. 3*C*).

### Synergistic effects of the other cellular kinases with CDK1 activity on lamin

**Figure 4:**
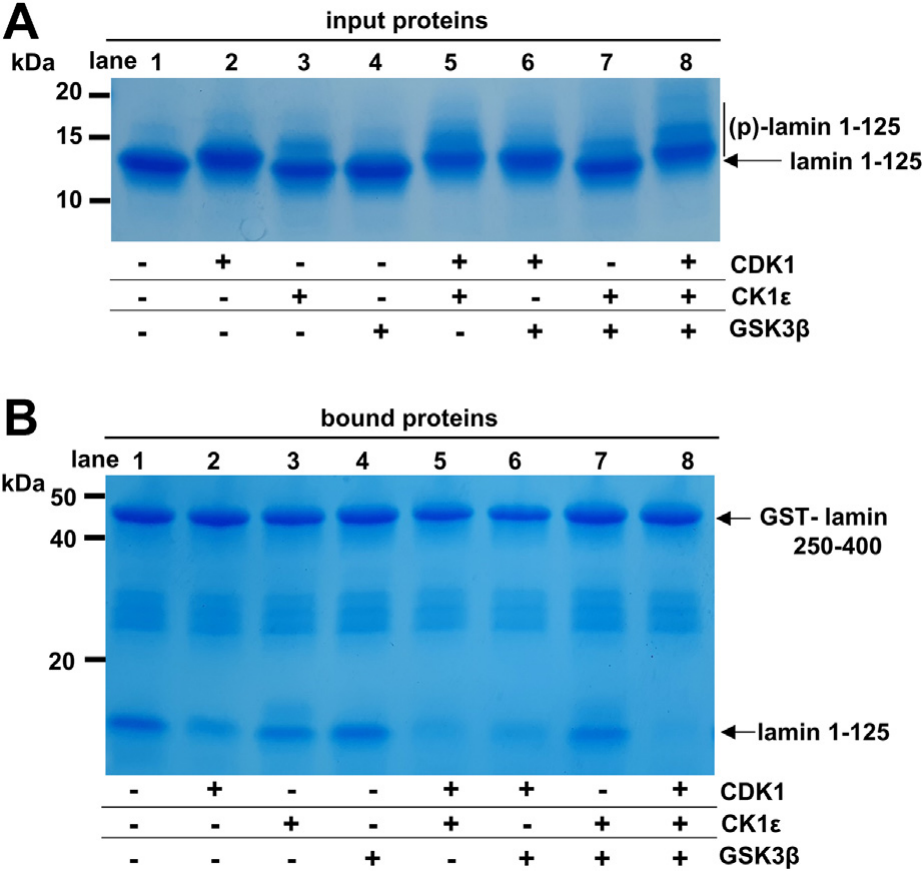
**The roles of phosphorylation by CK1 and GSK3β.***A,* the SDS-polyacrylamide gel was used to analyze the phosphorylation of input proteins of lamin 1 to 125 fragments by CDK1, CK1ε, and/or GSK3β. *B,* a GST pull-down assay was carried out to measure the ACN interaction using the GST-fused lamin coil 250 to 400. The lamin 1 to 125 fragment was phosphorylated by CDK1, CK1ε, and/or GSK3β and then loaded onto the GST-lamin 250 to 400–bound resin. The same amounts were used for CK1ε, GSK3β, and CDK1. Each sample was analyzed by SDS-PAGE. CDK1, cyclin-dependent kinase; CK1, casein kinase 1; GSK3β, glycogen synthase kinase 3β; GST, glutathione-*S*-transferase.

Cellular casein kinase 1 (CK1) and glycogen synthase kinase 3β (GSK3β) are activated in cells and act on preferential sites of the prephosphorylated Ser/Thr sites (pS/pT) (31, 35). CK1 can preferentially act on Ser or Thr of the sequence (pS/pT)XX(S/T), where the underlined Ser or Thr is the target site, and GSK3β can act on the sequence (S/T)XXX(pS/pT) (36, 37, 38). Because of the characteristic activities of CK1 and GSK3β, these two kinases increase the phosphorylation in adjacent regions of the prephosphorylation sites by the other kinases. To examine the cooperative action of CK1 and GSK3β with the CDK1–cyclin B complex, we incubated the recombinant CK1 and/or GSK3β proteins with the lamin N-terminal fragments (Fig. 4*A*). Without the CDK1 complex, CK1 and GSK3β treatment alone failed to abolish the ACN interaction. However, the cotreatment of CK1 and/or GSK3β with the CDK1 complex had synergistic effects on the attenuation of the ACN mode (Fig. 4*B*). These results suggest that the negative charges developed by phosphorylation are important in the attenuation of the ACN and further present a possibility in which the other cellular kinases amplify the phosphorylation signal by CDK1.

### The importance of the ionic interaction in the ACN binding

**Figure 5:**
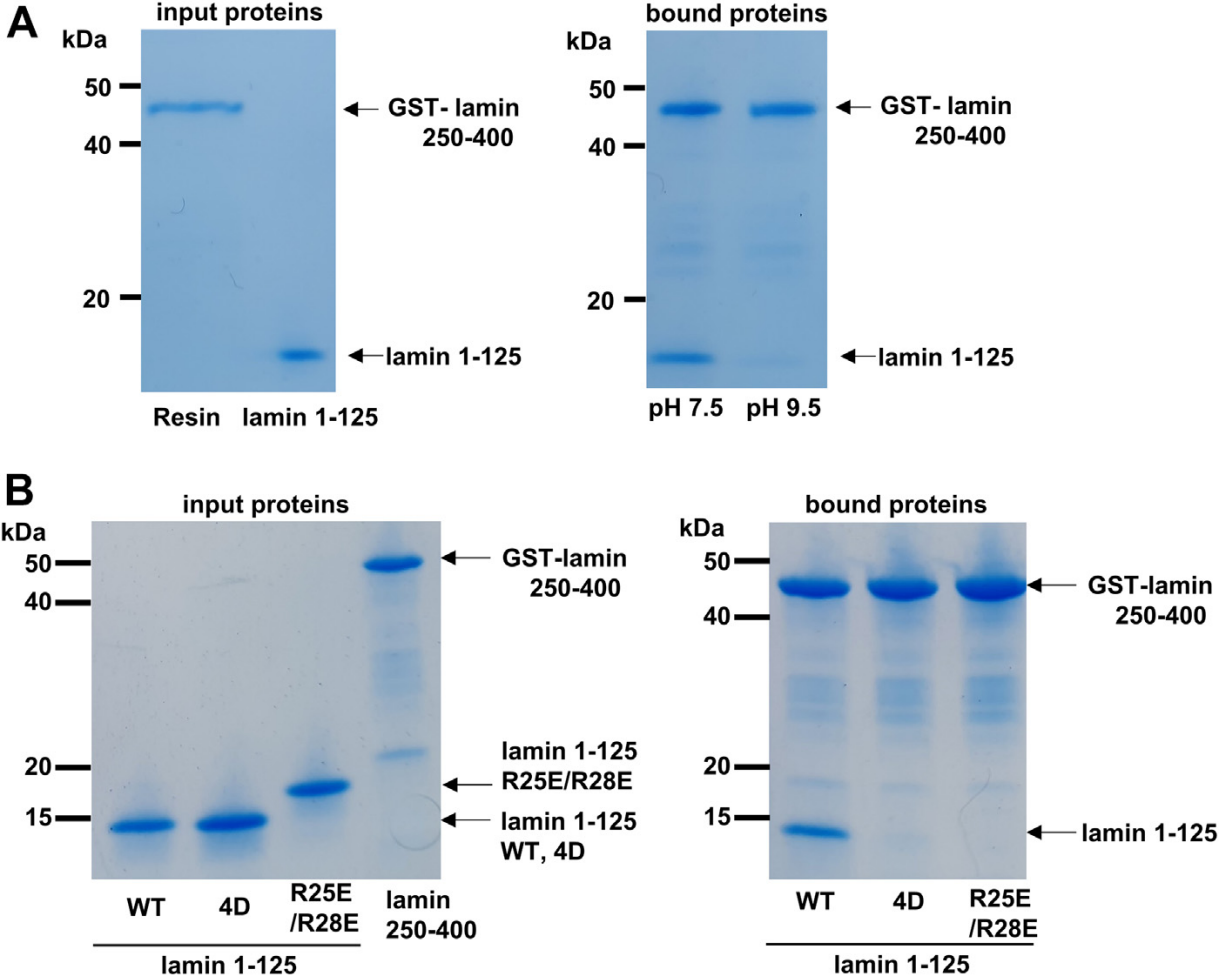
**Biochemical analysis of the ACN interaction using the GST-lamin coil 250 to 400 and the lamin 1 to 125 fragment.***A,* comparison of the ACN interactions between pH 7.5 and pH 9.5. GST-fused lamin 250 to 400 (GST-lamin coil 250–400) were covalently attached to the CNBr-activated resin, and then the lamin 1 to 125 fragment was incubated at the given pH. *B,* GST pull-down assay to test the importance of Arg25 and Arg28 in ACN interaction. All the proteins for the assay were prepared at the same concentration (input proteins, the *left panel*). The lamin 1 to 125 fragments (WT, 4D, or R25E/R28E) were incubated with GST-lamin 250 to 400–bound GSH-coupled resins, and then the bound proteins were analyzed by SDS-PAGE (bound proteins, the *right panel*). CNBr, cyanogens bromide; GST, glutathione-*S*-transferase.

To gain molecular insight into the phosphorylation-dependent dissociation of the ACN mode, we first tested whether the ionic interaction is important in the interaction between coil 1a and coil 2 regions. Since most ionic interactions are broken at high pH because of the absence of electrostatic charges at Lys and Arg, we measured the binding of coil 1a and coil 2 using the lamin 1 to 125 fragment and lamin 250 to 400 fragment at pH 9.5. To prevent the dissociation of the GST-fusion protein and GSH-resin at a high pH, we covalently linked the GST-lamin coil 250 to 400 fragment to the cyanogen bromide–activated resin. The results showed that the high pH condition abolished the binding of coil 1a and coil 2 (Fig. 5*A*), indicating that ionic interactions are involved in ACN bonding. We next noted two basic residues, Arg25 and Arg28, in the coil 1a region adjacent to the phosphorylation sites Thr19 and Ser22 in the chimeric structure of coil 1a (19). The R25E/R28E double mutation abolished the binding of coil 1a and coil 2, as shown in Figure 5*B*, suggesting the importance of the basic residues for the ACN.

## Discussion

Cell cycle–dependent morphological changes in the nucleus are essential for understanding life phenomena at the molecular level. The phosphorylation levels of lamins are elevated fourfold to sevenfold during mitosis than at interphase (39). In this study, we demonstrated that phosphorylation at the lamin N-terminal head region abolishes the interaction of the C-terminal part of coil 2 and coil 1a, which was previously designated as the ACN (18). We confirmed that the CDK1–cyclin B target sites Thr19 and Ser22 are critical in regulating the ACN mode. The multiple phosphorylation events in the head region additively contributed to the dissociation of the ACN without changing the coiled-coil propensity of the coil 1a region. In a previous study, the phosphorylation of S390 and S392 in the proximal C-terminal region before the immunoglobulin-like domain motif is also associated with the lamin filament disassemble (28). We also presented the possibility of the involvement of other kinases, such as CK1 and GSK3β, for multiple phosphorylation events. We further provided the molecular reason for the proper formation of the nuclear membrane in the laminopathy mutants.

**Figure 6:**
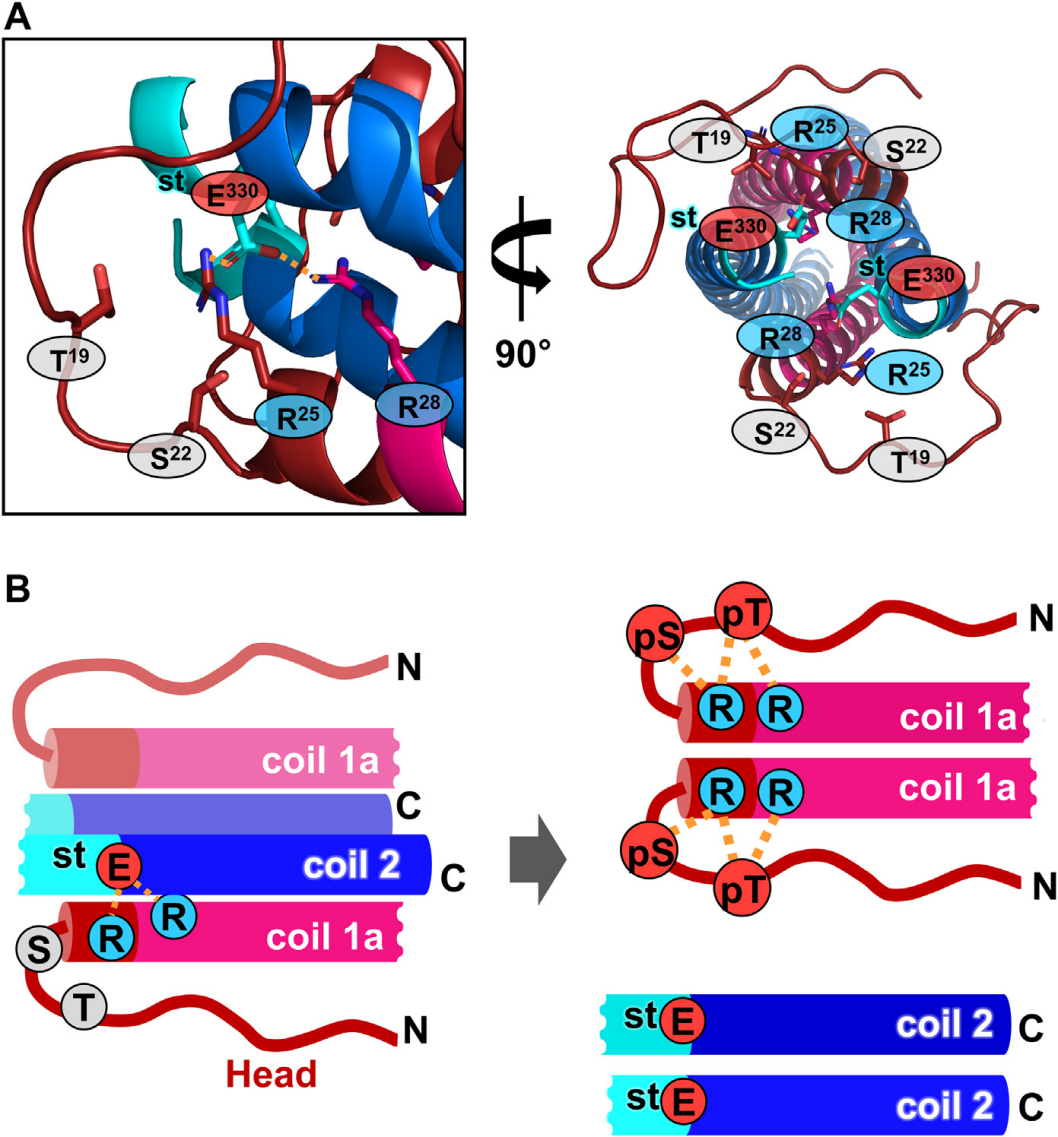
**A proposed mechanism of lamin depolymerization by phosphorylation.***A,* a model represents the ACN mode between coil 1a (*magenta*) and coil 2C (*blue*). The N-terminal head region (residues 1–27) was built by AlphaFold 2 (44). The four-helix bundle region (residues 28–80 and 327–385) was built from the Stalmans *et al*. (19) model based on the typical four-helix bundle structure. The combined structure was refined by GalaxyRefineComplex (40). The ionic interaction network (*yellow dotted lines*), consisting of Arg25, Arg28, and stGlu330 (Glu330 at Stutter) (*stick representations*), was found near Thr19 and Ser22 (*stick representations*). The *circle* is colored to indicate the charge of residue (*red*, negatively charged residue; *blue*, positively charged residue; and *gray*, Thr19 and Ser22 residues). *B,* a proposed mechanism of lamin depolymerization. During interphase, the ACN is stabilized by the ionic interaction network (*left*). CDK1 activity during mitosis builds up the negative charges at the phosphorylation sites Thr19 and Ser22 (*right*). Then, the negative charges may compete and disrupt the ionic interaction network between coil 1a and the C-terminal region of coil 2, leading to the disassembly of the lamin filaments. CDK1, cyclin-dependent kinase 1.

Then, we wanted to determine how phosphorylation in the N-terminal head domain promotes the dissociation of the binding between coil 1a and the C-terminal part of coil 2. We noted the complex model of coil 1a and a C-terminal part of coil 2, built by Stalmans *et al.* (19), based on the individual proteins’ structures (Fig. 6). Stalmans *et al.* (19) made a parallel four-helix bundle model, which matched the cross-linking mass analysis by Makarov *et al.* (18). Our previous results further supported the four-helix bundle formation that the separation of coil 1a and the C-terminal part of coil 2 was required (33). The separation of the coiled-coils was needed for the structural transition from two coiled-coil dimers to the complex. To better estimate the role of phosphorylation in the N-terminal head region, this study created a similar complex model of the ACN mode, including the N-terminal head region. This model showed that the N-terminal head region folds back to coil 1a, supported by the cross-linking mass analysis (18). According to the model refined by GalaxyRefineComplex (40), Arg25 and Arg28 form an ionic interaction network with the conserved Glu330 residue of the stutter region in the central cavity of the four-helix bundle (Fig. 6*A*). This structural arrangement stabilizes the four-helix bundle structure at its end.

Based on these findings, we propose a phosphorylation-dependent dissociation mechanism for the lamin filaments. The lamin filaments are stabilized by the intramolecular ACN mode between coil 1a and coil 2 *via* the Glu330 and Arg25–Arg28 residues during the interphase (Fig. 6*B*, *left*). In the mitosis phase, the activated CDK1–cyclin B complex phosphorylates the N-terminal head region, forming an intramolecular ionic interaction with the basic residues of the coil 1a parts. These intramolecular ionic interactions disrupt the intermolecular ionic interaction network with the Glu residues at the end of the four-helix bundle structure (Fig. 6*B*, *right*). Thus, coil 1a in our model serves as a pivot in the ACN mode between forming the intermolecular ionic interaction with coil 2 and the intramolecular interaction with the phosphorylated head region. This disassembly of the ACN mode would finally trigger the depolymerization of the lamin filaments.

The cell cycle–dependent morphological change in the nuclear envelope is regulated by the phosphorylation state of the N-terminal head region of lamin. To understand its precise mechanism, we need to understand the biochemical roles of phosphorylation in the context of the lamin complex structure. This study has revealed the biochemical roles of phosphorylation in the depolymerization of lamin filaments. Our results will help elucidate the cell cycle–dependent regulation of the nuclear shape, which is a fundamental phenomenon in all eukaryotic cells.

## Experimental procedures

### Expression and purification of proteins

WT lamin 1 to 125 fragment (human lamin A/C, residues 1–125) and mutant forms (T19A, S22A, T19A/S22A, S22D, T19D/S22D, T10D/S12D/T19D/S22D, and R25E/R28E) with an N-terminal hexaHis-tag were expressed in *Escherichia coli* as previously described (33). Human lamin A/C 250 to 400 (coil 2C-containing fragment in the main text) was expressed in *E. coli* as an N-terminal GST-fusion protein. Cells were cultured in a Terrific broth medium at 37 °C overnight, induced with 0.5 mM IPTG, and grown for another 6 h. After the cells were harvested, they were resuspended in lysis buffer (20 mM Tris–HCl [pH 8.0], 150 mM NaCl, and 2 mM β-mercaptoethanol) and then disrupted using a French press (23 kpsi). The cell debris was removed by centrifugation, and the supernatant was loaded onto glutathione–agarose resin (GE Healthcare) preincubated with lysis buffer. The target protein was eluted with lysis buffer supplemented with 20 mM reduced GSH. For further purification, the target protein was loaded onto a HiTrap Q column (GE Healthcare) and eluted with 300 mM NaCl. The purified proteins were desalted using a HiPrep 16/60 desalting column (GE Healthcare) in 20 mM Tris–HCl (pH 7.5) and 50 mM NaCl.

The catalytic domain (residues 1–319) of the human CK1ϵ catalytic domain with the C-terminal hexaHis-tag was expressed in *E. coli* (41). The bacterial cells were cultured in a Luria–Bertani medium at 37 °C until an absorbance of 0.7 at 600 nm, and the protein was expressed by induction with 0.5 mM IPTG for 6 h. After the cells were harvested, they were resuspended in lysis buffer (20 mM Tris–HCl [pH 7.5], 150 mM NaCl, and 2 mM β-mercaptoethanol) and then disrupted using a French press (23 kpsi) and cleared by centrifugation. The supernatant was loaded onto nickel–nitrilotriacetic acid resin (GE Healthcare) preincubated in lysis buffer. CK1ε was eluted with lysis buffer supplemented with 250 mM imidazole and 0.5 mM EDTA. The eluted protein was loaded on a HiLoad 16/600 column (GE Healthcare) with 20 mM Tris–HCl (pH 7.5), 150 mM NaCl, 0.5 mM EDTA, and 2 mM β-mercaptoethanol. The recombinant GSK3β protein was obtained as outlined previously (42).

### Phosphorylation of the lamin N-terminal fragments

One hundred micrograms of each lamin fragment were phosphorylated by 1 μg of the CDK1–cyclin B complex protein (catalog no.: PV3292; Thermo Fisher Scientific) or CK1ε or GSK3β for 2 h at 30 °C in 200 μl of 20 mM Tris–HCl, pH 7.5, buffer containing 50 mM NaCl, 20 mM MgCl_2_, 0.5 mM EDTA, 1 mM ATP, and 2 mM DTT. The reaction was stopped by adding 10 μM flavopiridol (CDK1 inhibitor; Selleckchem) to the reaction mixture.

### GST pull-down assays

The phosphorylated or nonphosphorylated lamin N-terminal fragments (20 μM) and GST-fused coil 2C fragment (5 μM) were incubated in 300 μl of 20 mM Tris–HCl (pH 7.5) buffer containing 50 mM NaCl with GSH–agarose resin for 1 h at room temperature. The resins were washed with 50 mM Tris–HCl (pH 7.5) buffer containing 50 mM NaCl and eluted with buffer supplemented with 20 mM reduced GSH. The samples were analyzed by a 4 to 20% gradient SDS-polyacrylamide gel (Bio-Rad) or a Phos-tag containing SDS-polyacrylamide gel (Wako).

### Lamin coil 2–conjugated Sepharose pull-down assay

The GST-fused lamin coil 2C–containing fragment was conjugated with the resin using cyanogen bromide–activated Sepharose (GE Healthcare), resulting in lamin coil 2–conjugated Sepharose. The lamin coil 2–conjugated Sepharose was washed with 20 mM Bis–Tris propane (pH 7.5) buffer or 20 mM Bis–Tris propane (pH 9.5) buffer. Each washing buffer contained 50 mM NaCl. The lamin 1 to 125 fragment (50 μg) was incubated with lamin coil 2–conjugated Sepharose for 1 h at room temperature. The Sepharose resins were washed with washing buffer and loaded on SDS-PAGE gels (Bio-Rad).

### CD

The purified WT and mutant lamin 1 to 125 fragments (1 mg/ml) were dialyzed against PBS. Then, the CD spectra were recorded by UV from 190 to 260 nm in the CD detector Chirascan Plus (Applied Photophysics). The protein secondary structure elements were analyzed using CDNN software (CD analysis using neural networks) (Applied Photophysics) (43).

### Lamin complex modeling

We obtained the N-terminal flexible head region structure from the structure predicted by AlphaFold 2 Colab (44) using the lamin sequence (residues 1–80). We built the parallel four-helix bundle structure of coil 1a (residues 28–67) and coil 2C (residues 327–385) using a typical parallel four-helix bundle structure (Protein Data Bank code: 1G1J) as the structural template. We changed the residues into the sequences of coil 1a and coil 2C based on the sequence alignment by referring to the model of Stalman *et al*. (19). We combined the N-terminal head region with the parallel four-helix bundle structure and then refined the combined structure by GalaxyRefineComplex (40).

## Data availability

All data are contained within the article.

## Supporting information

This article contains supporting information (30, 43).

## Conflict of interest

The authors declare that they have no conflicts of interest with the contents of this article.

## Supporting information

supporting informations
