# Supplementary material for: Cyclin-dependent kinase 1 depolymerizes nuclear lamin filaments by disrupting the head-to-tail interaction of the lamin central rod domain
Source: J Biol Chem. 2022 Jul 15;298(9):102256. doi: 10.1016/j.jbc.2022.102256 (PMC9400090; doi:10.1016/j.jbc.2022.102256)
Supplement: supporting informations [file mmc1.pdf]

Cyclin-Dependent Kinase 1 depolymerizes nuclear lamin filaments by disrupting the head-to-tail interaction of the lamin central rod domain.

Soyeon Jeong[1], Jinsook Ahn[1], Inseong Jo[1], So-Mi Kang[2], Bum-Joon Park[2], Hyun-Soo Cho[3], Yong-Hak Kim[4], and Nam-Chul Ha[1*]

[1]Department of Agricultural Biotechnology, Center for Food and Bioconvergence, and Research Institute for Agriculture and Life Sciences, CALS, Seoul National University, Seoul 08826, Republic of Korea

[2]Department of Molecular Biology, College of Natural Science, Pusan National University, Busan 46241, Republic of Korea

[3]Department of Systems Biology and Division of Life Sciences, Yonsei University, 50 Yonsei-ro, Seodaemun-gu, Seoul, 03722, Republic of Korea

[4]Department of Microbiology, Catholic University of Daegu School of Medicine, Daegu, Republic of Korea

[*]To whom correspondence should be addressed: hanc210@snu.ac.kr

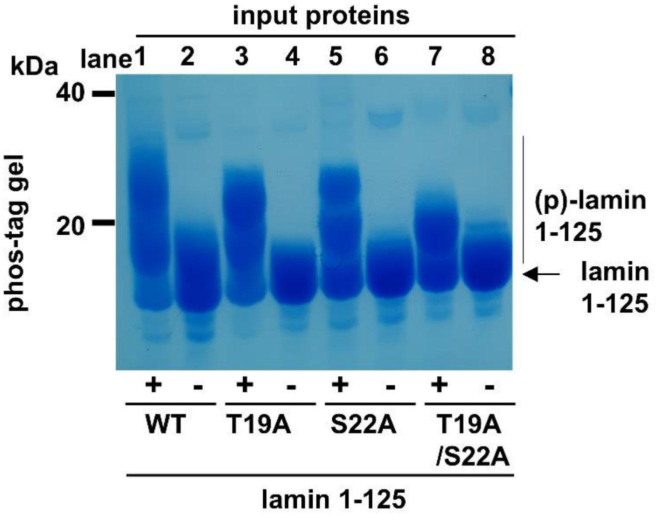

Supplementary Figure 1. Phosphorylation of the wild-type and mutant lamin 125 fragments. The lamin 125 fragments harboring the mutations T19A, S22A, and T19A/S22A were phosphorylated by treating the CDK1/cyclin B complex (0.5 μg/ml) for 2 hrs at room temperature. A portion of the resulting proteins was subjected to SDS–PAGE, and the rest of the proteins were used in the GST pull-down assay shown in Fig. 2D. The phosphorylated protein bands ((p)-lamin 125; Lanes 1, 3, 5, and 7) are at slightly higher positions than the nonphosphorylated protein bands (Lanes 2, 4, 6, and 8).

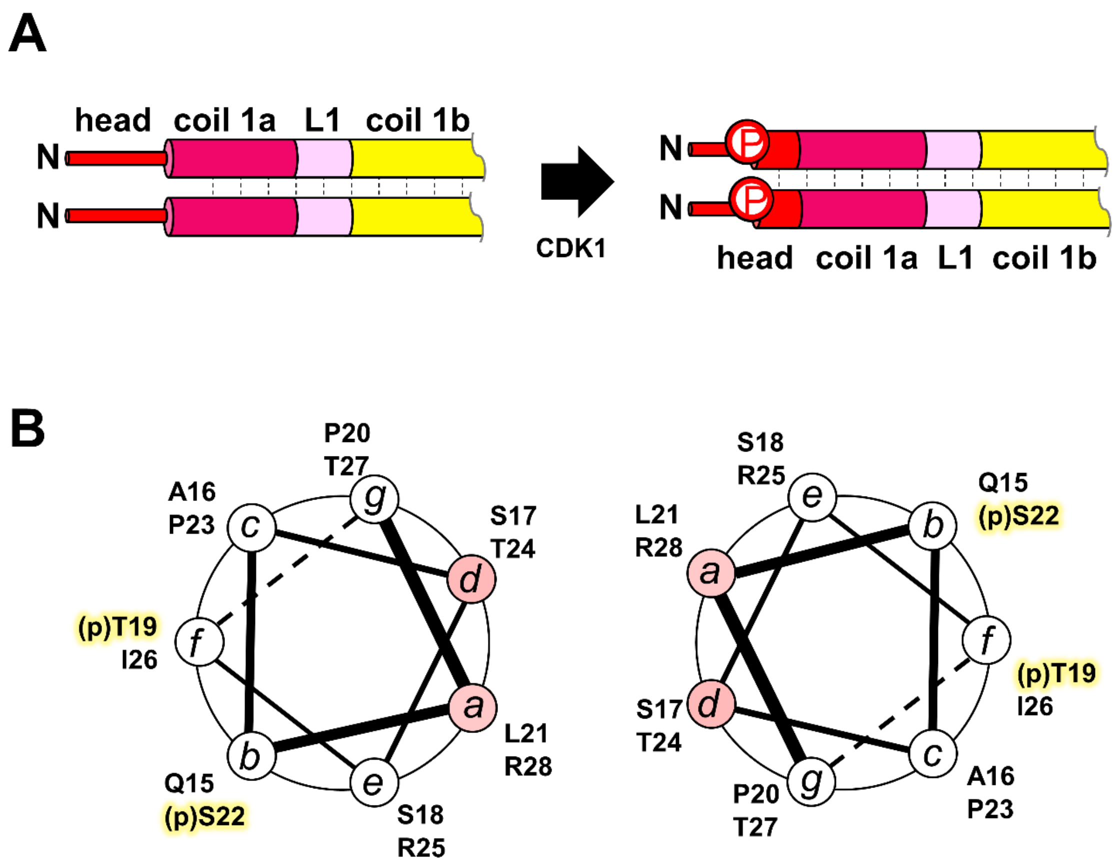

Supplementary figure 2. A hypothesis when the Thr19 and S22 phosphorylated

A. A hypothesis representing the phosphorylation-dependent change in the coiled-coil propensity of the coil 1a region is presented with a schematic drawing of the lamin 1-125 fragment. P in the circles indicates the phosphorylated Ser/Thr. The thick cylinders represent the α-helices and the thin cylinders represent the unstructured region. The coiled-coil interactions are represented as dotted lines between the α-helical regions. Note that the phosphorylation expands the coil 1a α-helical regions to the N-terminal head region.

B. The diagram using the heptad positions of the primary sequence and helical wheel plot. The residues are marked next to the corresponding heptad repeat position and the position described in italic (*a* to *g*). The position of *a* and *d* represented the red circle, and phosphorylation residues were colored yellow.

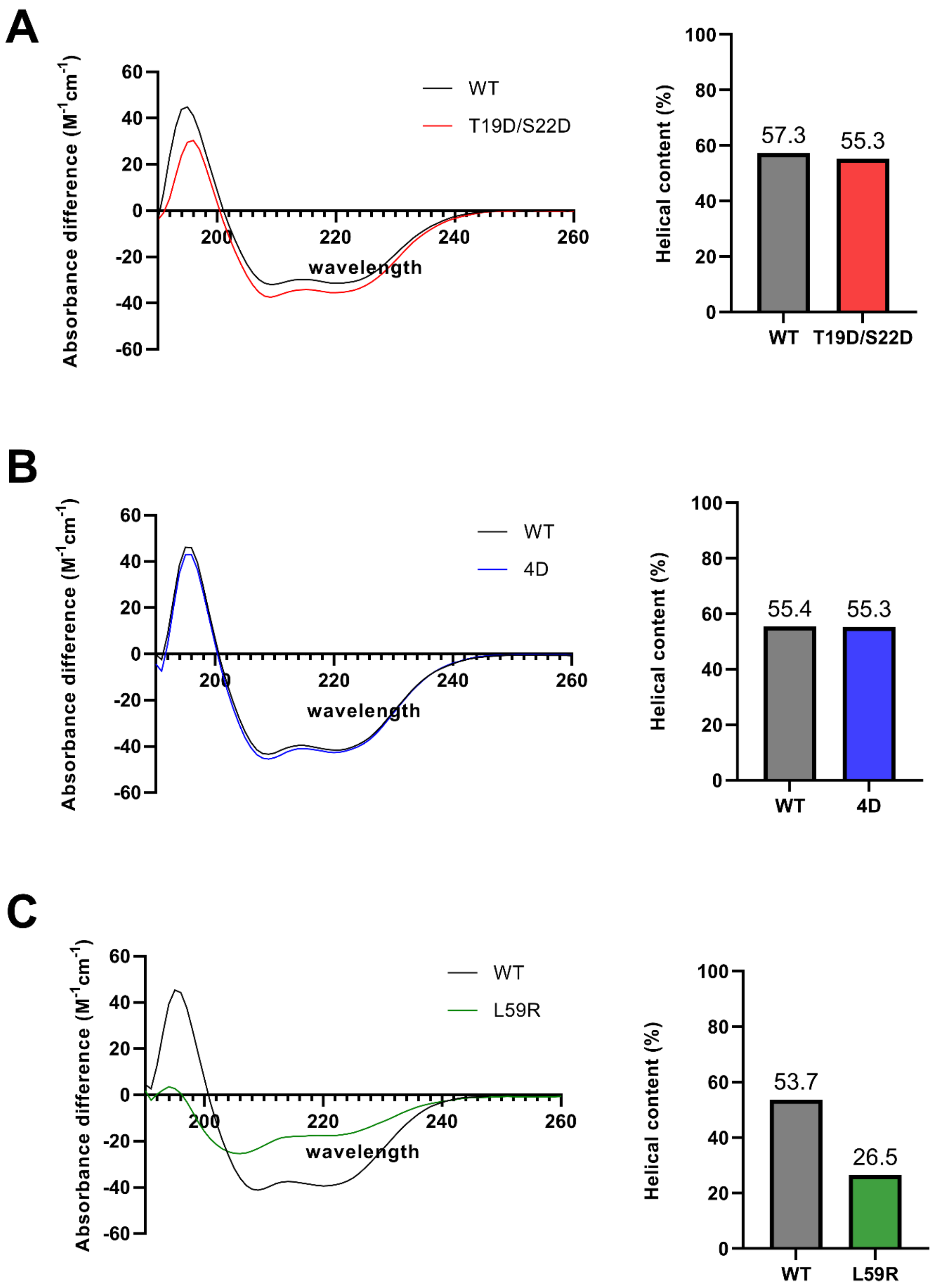
A
Absorbance difference (M-1cm-1)
wavelength
WT
T19D/S22D
Helical content (%)
57.3
55.3
WT
T19D/S22D
B
Absorbance difference (M-1cm-1)
wavelength
WT
4D
Helical content (%)
55.4
55.3
WT
4D
C
Absorbance difference (M-1cm-1)
wavelength
WT
L59R
Helical content (%)
53.7
26.5
WT
L59R

Supplementary Figure 3. The CD spectra of the wild-type and mutant lamin 125 fragments. A. The CD spectra (left) of the wild-type (black) and T19D/S22D mutant (red) lamin 125 fragments (1 mg/ml) and their helical contents were calculated from the CD spectra (right). B. The CD spectra (left) of the wild-type and 4D mutant (blue) lamin 125 fragments (1 mg/ml) and their helical contents were calculated from the CD spectra (right). C. The CD spectra (left) of the wild-type and L59R mutant (green) lamin 125 fragments (1 mg/ml) and their helical contents were calculated from the CD spectra (right).
